# Inadvertent side effects of fixed lingual retainers: An in vitro study

**DOI:** 10.1007/s00056-022-00432-4

**Published:** 2022-11-11

**Authors:** Marlen Seide, Teresa Kruse, Isabelle Graf, Christoph Bourauel, Bernd G. Lapatki, Rudolf Jäger, Bert Braumann

**Affiliations:** 1 Faculty of Medicine and University Hospital Cologne, Department for Orthodontics, University of Cologne, Kerpener Street 32, 50931 Cologne, Germany; 2 Oral Technology, University Hospital Bonn, Welschnonnenstraße 17, 53111 Bonn, Germany; 3 Department of Orthodontics, Center of Dentistry, University of Ulm, Albert-Einstein-Allee 11, 89081 Ulm, Germany

**Keywords:** Triple-stranded retainer wires, Posttreatment changes, X‑effect, Unwanted tooth movement, Long-term stability, Dreifach verseilte Retainerdrähte, Posttherapeutische Veränderungen, X‑Effekt, Nichterwünschte Zahnbewegung, Langzeitstabilität

## Abstract

**Purpose:**

To better understand the side effects of fixed lingual retainers by means of an in vitro study in a two-tooth model determining the three-dimensional (3D) force–moment components acting at adjacent teeth combined with different composite–wire interfaces.

**Methods:**

Triple-stranded round retainer wires were embedded in cured disks of flowable composite. At one side the composite–wire interface was untreated and checked to be absolutely fix. At the other side the composite–wire interface was configured as either an isolated compound with (1) petroleum jelly coating, or an adhered compound with (2) no manipulation, (3) ethanol degreasing or (4) ethanol degreasing and rectangular bending of the wire ends. The 3D force–moment components were registered, while the intertooth distance was increased in steps of 0.01 mm leading to increasing tension of the wire. Measurements were repeated after artificially aging the specimens.

**Results:**

Retainer wire specimens with adhered compound (2, 3, 4) showed negative vestibulo-oral moments ranging maximally each between −0.3 and −0.9 Nmm in opposite direction to positive moments of 1.9 Nmm for specimens with isolated compound 1. Significant tipping moments occurred in the group with isolated compound at lower forces than in those groups with adhered compound. Similar effects were observed after artificial aging.

**Conclusion:**

Side effects emerge under specific circumstances: an altered adhesive compound combined with the presence of oral forces. Compounds with lost adhesion at the composite–wire interface showed rotational moments in the direction of the wire windings even during low tensile forces similar to those that may occur in clinical settings. Opposite rotational moments leading to unwinding of the wire may occur in cases with adhered compounds at higher tensile forces. Utilization of round triple-stranded retainer wires without bent ends are of higher risk to induce inadvertent side effects.

## Abstract (de)

**Ziel:**

Um Nebenwirkungen festsitzender Lingualretainer besser zu verstehen, wurde eine In-vitro-Studie an einem Zweizahnmodell durchgeführt, bei der die dreidimensionalen (3-D) Kraft-Moment-Komponenten bestimmt wurden, die auf aneinandergrenzende Zähne in Kombination mit verschiedenen Komposit-Draht-Grenzflächen wirken.

**Methoden:**

Dreifach verseilte runde Retainerdrähte wurden in ausgehärtete Scheiben aus fließfähigem Komposit eingebettet. Auf der einen Seite wurde die Komposit-Draht-Grenzfläche unbearbeitet belassen und auf absolute Festigkeit geprüft. Auf der anderen Seite wurde die Komposit-Draht-Grenzfläche entweder mit (1) Petrolatum isoliert oder als feste Verbindung (2) ohne weitere Bearbeitung, (3) mit Ethanol-Reinigung oder (4) mit Ethanol-Reinigung und rechtwinkligem Umbiegen der Drahtenden gestaltet. Die Kraft-Moment-Komponenten wurden dreidimensional erfasst, während der Zwischenzahnabstand in Schritten von 0,01 mm vergrößert wurde, was zu einer zunehmenden Spannung des Drahtes führte. Die Messungen wurden nach einer künstlichen Alterung der Proben wiederholt.

**Ergebnisse:**

Drähte mit festem Kunststoffhaftverbund (2, 3 und 4) zeigten negative Drehmomente in vestibulooraler Richtung mit Maximalwerten zwischen −0,3 und −0,9 Nmm. Proben mit gelöstem Verbund (1) zeigten ein entgegengesetztes positives Drehmoment mit Maximalwerten von 1,9 Nmm. Relevante „tipping moments“ traten in der Gruppe mit isoliertem Verbund bei geringeren Kräften auf als in den Gruppen mit adhärentem Verbund. Vergleichbare Effekte wurden an künstlich gealterten Probenkörpern beobachtet.

**Schlussfolgerung:**

Nebenwirkungen treten unter spezifischen Bedingungen, wie eine veränderte Haftverbindung in Kombination mit dem Einwirken von oralen Kräften, auf. Verbindungen mit gelöstem Haftverbund an der Grenzfläche zwischen Komposit und Draht zeigten Drehmomente in Richtung der Drahtwindungen. Dies war selbst bei geringen Zugkräften, ähnlich denen, die in klinischen Situationen entstehen können, zu beobachten. Umgekehrte Drehmomente, die zu einem Entwinden des Drahtes führen, können bei fester Verbindung und höheren Zugkräften auftreten. Die Verwendung von runden, dreifach verseilten Retainerdrähten ohne umgebogene Enden stellen ein erhöhtes Risiko für das Auftreten von nicht beabsichtigten Nebenwirkungen dar.

## Introduction

Long-term stability after active tooth movement is substantial and retention is a key part of the orthodontic treatment. In recent years, the number of inserted fixed retainers has increased as they are very effective and require less patient compliance than removable retainers [15, 17]. Segmental frontal wires with different shape, type (i.e., solid, multistranded or braided), material and dimensions can be bonded to all 6 anterior teeth or only to the canines [2, 3]. The different designs of fixed lingual retainers show varying advantages in terms of periodontal effects and survival rates [8, 12, 26]. Differences have also been discussed with regard to relapse and inadvertent tooth movements [23].

Despite precautions taken, the tendency of unwanted tooth movement cannot be avoided completely. Changes may occur due to relapse towards initial tooth malposition or due to post pubertal growth and aging [28, 29]. Damaged fixed retainers cannot completely withstand such influences. Retainer failure mostly occur within the first 3–6 months after insertion, with significantly less failure after one year. The overall rate of bonding failure reported in the literature ranges between 30–40% [1, 6]. Incorrect refixation of the retainer may lead to an unintentional activation of the retainer wire inducing even more unwanted tooth movements. Masticatory forces deforming the wire or incorrect handling during the first bonding may similarly lead to an active retainer wire. This is a frequent complication in orthodontic practice [17, 23].

**Fig. 1:**
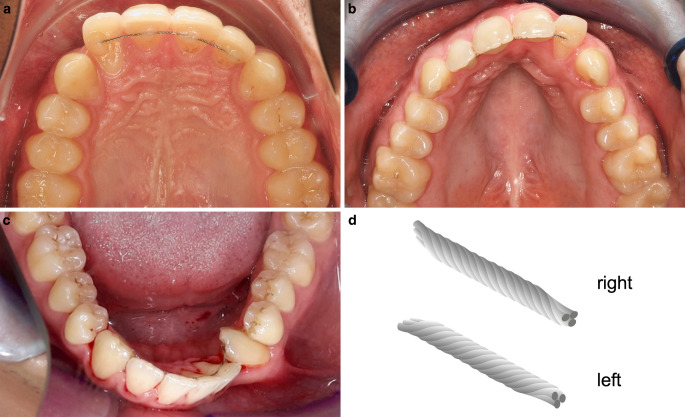
Exemplary clinical cases with inadvertent tooth movements under fixed retention. A recognizable pattern of the x‑effect is shown by isolated labial tipping of the upper right lateral incisor explained by torqueing this tooth around the axis of a round triple-stranded retainer wire with right-handed windings (**a**). The same pattern is observable with a labial tipping of the upper left lateral incisor torqueing around the axis of a round triple-stranded retainer wire with left-handed windings (**b**). Severe malposition of the whole frontal segment consisting of severe lingual tipping of the lower left canine and labial tipping of the incisor segment is characteristic for the twist effect (**c**). Triple-stranded round retainer wires are available with right-handed windings (*right*) and left-handed windings (*left*; **d**) Exemplarische klinische Fälle mit nicht beabsichtigten Zahnbewegungen unter festsitzender Retention. Typisches Bewegungsmuster des X‑Effektes mit isoliertem Labialkippen des rechten seitlichen Schneidezahnes, erklärbar durch eine Torquebewegung des Zahnes um die Achse des runden, dreifach verseilten rechtsgewundenen Retainerdrahtes (**a**). Ähnliches Bewegungsmuster mit Labialkippen des seitlichen linken Schneidezahnes und Torquebewegung des Zahnes um die Achse des runden, dreifach verseilten linksgewundenen Retainerdrahtes (**b**). Ausgeprägte Fehlstellungen im gesamten Frontzahnsegment mit starker Lingualkippung des unteren linken Eckzahns und Labialkippung des Frontzahnsegmentes, charakteristisch für den Twist-Effekt (**c**). Dreifach verseilte runde Retainerdrähte sind rechtsgewunden („right“) und linksgewunden („left“) erhältlich (**d**)

While the latter effects are plausible, the explanation for active tooth movements under fixed retention occurring even several years after insertion without particular impact and without visible damage of the fixed retainer is still unclear. The etiology of this phenomenon, named x‑effect or twist effect, may be multifactorial. Characteristic changes are described as rotational movements of single teeth (x-effect, Fig. 1a,b) or of the whole incisor segment with opposite torque of the canines (twist effect, Fig. 1c) [9, 14, 18, 30]. Such effects may be expressed as mild malposition to more severe malposition that require an orthodontic follow-up treatment—possibly even in cooperation with a periodontist [23]. Such inadvertent tooth movements may be at least partly caused by the retainer itself without a relation to the former malocclusion [9, 18, 30]. Mechanical properties of the wire may be crucial [14], especially in cases where an obvious explanation such as wrong bonding or fracture of the retainer wire can be excluded. It is largely unclear if production-related characteristics of the retainer wire are decisive, such as differences in right- or lefthanded windings of a stranded wire (Fig. 1d).

The extent to which forces exerted by the soft-tissue during chewing, swallowing and dysfunction (e.g., tongue thrust) are involved in the x‑ or twist effect is not conclusively clarified [4, 25]. Actually, such forces may lead to relative movements between adjacent teeth and therefore tension of the retainer wire and the wire–composite interface. Such tensile forces may be facilitated by the elasticity of the periodontal ligament [16]. A previous study found a correlation between affected patients and existing oral dysfunctions or habits [12]. Hence, oro-vestibular-directed forces onto the teeth may be (at least partly) responsible for inadvertent tooth movements of the retainer segment.

This study intends to explain inadvertent tooth movements of teeth connected by a fixed retainer wire in an experimental two-tooth model. Following the analogy to a bolt and nut with a loosened nut rotating in the direction of the windings during tensile forces, we assumed that three conditions may hold for the development of inadvertent moment, which clinically manifests in the x‑effect. First, the adhesive compound between the retainer wire and the composite must be altered to allow a certain degree of rotational tooth movement. Second, a certain horizontal force component is required to create a rotational moment. Third, the retainer has to be a stranded wire. These three conditions may lead to a situation where the terminal teeth rotate around the axis of the retainer wire. The aim of this study was to experimentally investigate under which circumstances retainer wires may generate active moments explaining the observed inadvertent tooth movements under fixed retention.

## Methods

### Experimental retainer wire loading

**Fig. 2:**
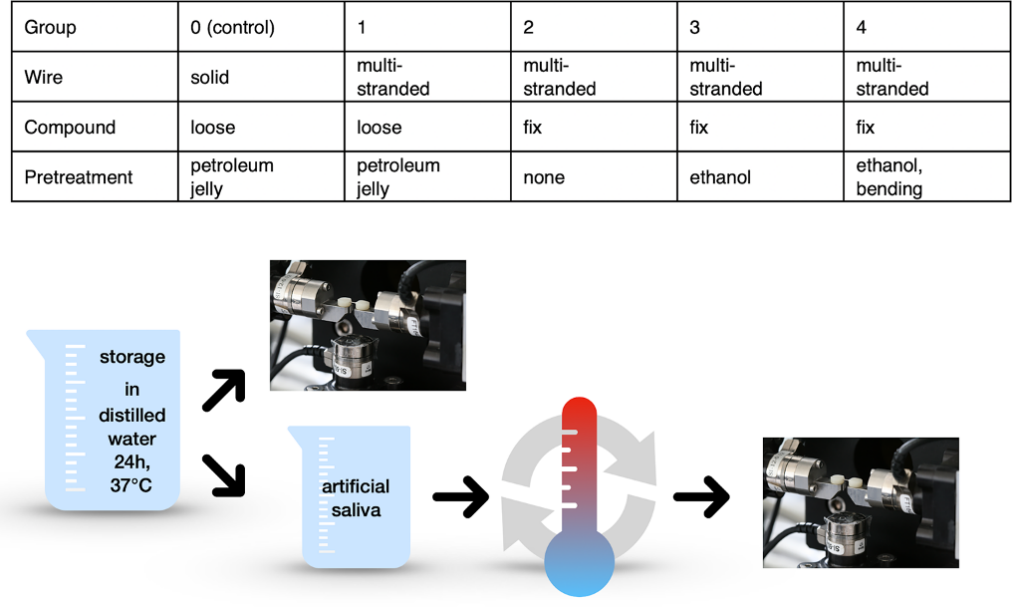
Group classification and treatment protocol. Groups with loose adhesive compound: Control group 0 and group 1. Groups with a fixed adhesive compound: groups 2, 3 and 4. All samples were stored in a humid environment for 24 h to reach final curing hardness of the composite. Half of the specimens of each group underwent tensile tests, while the other half was artificially aged before testing Gruppeneinteilung und Versuchsaufbau. Gruppen mit gelöstem Haftverbund: Kontrollgruppe 0 und Gruppe 1. Gruppen mit festem Haftverbund: Gruppen 2, 3 und 4. Alle Proben wurden für 24 h in feuchtem Milieu gelagert, um eine ausreichende Endhärte des Komposites zu gewährleisten. Die Hälfte der Proben jeder Gruppe wurde im Zugversuch belastet, die andere Hälfte wurde zuvor künstlich gealtert

The combination of a retainer wire of 15 mm length and cured flowable composite coating at both ends served as a two-tooth model (Grandio Flow, VOCO GmbH, Cuxhaven, Germany, cured with UV lamp for 20 s each). In order to identify which mechanical conditions induce inadvertent forces or moments, we replicated this model with an altered composite–wire interface in one of the teeth. The uncoated central part of the wire with a span of 3 mm corresponded to the distance between the adhesive points of two adjacent teeth connected with a fixed retainer. As a control we used the same setup in combination with a solid round stainless steel wire (diameter 0.45 mm, Lee Orthodontic Wire, Lee Pharmaceuticals, South El Monte, CA, USA), To allow the composite to reach final curing hardness, all specimens were stored in distilled water at 37 °C for 24 h (Fig. 2).

To obtain a fixed (adhered) or loose (isolated) composite–wire interface on the left side of each specimen, the wires on this side were preconditioned in different ways before bonding. In the control group (group 0) and in group 1, petroleum jelly coating enabled a relative movement between the wire and the composite; in group 2, the wire was not pretreated and in group 3 the wire was degreased with ethanol resulting in maximally strong bonding. The wire ends in group 4 were additionally bent rectangularly further stabilizing the firm wire–composite connection. The composite–wire interface on the right side of each specimen was checked to be absolutely fixed. With exception of the control group (0), triple-stranded stainless steel retainer wires (diameter 0.45 mm dentaflex®, Dentaurum GmbH & Co. KG, Ispringen, Germany) were used in groups 1–4.

We examined 20 samples per group leading to 100 samples in total. To investigate the effect of artificial aging, half of the specimens of each group (10 samples each) underwent a simulation of a 2-year retention time in an oral environment. First, the samples were stored in artificial saliva (Fusayama/Meyer, Sigma-Aldrich Chemie GmbH, Taufkirchen, Germany) for 24 h at 37 °C. Second, 5000 cycles of thermocycling between 5 ° and 55 °C were performed (Fig. 2).

**Fig. 3:**
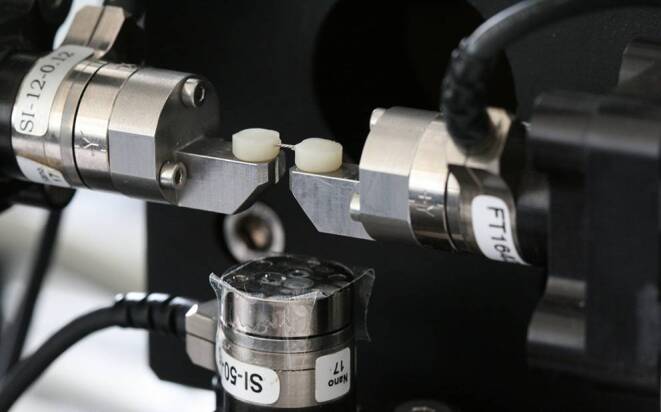
Specimen installed in the testing machine consisting of translational and rotational stages (PLS85; PI miCos, Eschbach, Germany) equipped with 3D F/M sensors (Nano 17, ATI Industrial Automation, Apex, NC, USA) Probenkörper im Messaufbau, bestehend aus Translations- und Rotationseinheiten (PLS85; PI miCos, Eschbach) sowie 3‑D F/M-Sensoren (Nano 17, ATI Industrial Automation, Apex/NC, USA)

Experiments were performed in the biomechanics lab of the orthodontic department at Ulm University. Relative movements between the wire and the composite due to oral forces were simulated in vitro by application of a tensile force on the retainer wire generated by a linear translation stage (PLS85; PI miCos, Eschbach, Germany). The composite ends of each specimen were firmly fixed to both crossheads, as illustrated in Fig. 3. In all tensile tests, both crossheads were moved apart in steps of 0.01 mm with a speed of 1 step per second leading to tension of the retainer wire. Total movement consisted of 15 steps resulting in 0.15 mm tension of the tested wires.

A rotational moment Mx around the wire axis (x) was expected to cause an inclination change of a tooth in labio-oral direction in vivo and, therefore, was the main force–moment component of interest. In addition, the tensile force Fx between the two teeth was regarded as a causative factor for Mx. Both variables were determined by averaging corresponding Mx and Fx components registered at both model teeth, because in a perfectly aligned two-tooth model, Mx and Fx values measured at both teeth should be of equal magnitude but opposite sign due to the equilibrium of forces and moments in a static situation. Force and moment components were measured with a resolution of 0.003 N or 0.016 Nmm by commercially available 3D force/moment sensors (Nano 17, ATI Industrial Automation, Apex, NC, USA) integrated into the measurement setup. All specimens were moisturized with distilled water to simulate the oral environment while measuring.

### Data analysis

Data were acquired using LabViEW software (National Instruments, Austin, TX, USA) and analyzed using the software MATLAB (The Math Works Inc., Natick, MA, USA). Corresponding algorithms were developed in-house. Incremental axial tension of the retainer wire resulted in 15 measurement points between neutral position and maximum deflection, ensuring sufficient resolution of the load–deflection curves. Data measured at the wire–composite interface for the 10 non-aged and aged specimens each showed a normal distribution. They were averaged by determining mean force and moment values.

To statistically evaluate the differences to zero, measured moments of all triple-stranded wires (group 1–4) were analyzed using a one-sample student’s t‑test. Due to multiple comparisons, the four statistical tests were Bonferroni-corrected. To assess differences between groups, two-sample student’s t‑tests were performed. The six group comparisons also were Bonferroni-corrected. *P*-values smaller than 0.05 were considered as statistically significant. All statistical analyses were performed in Microsoft® Excel for Mac, version 16.41 (Microsoft Corporation, Redmond, WA, USA).

## Results

Varying forces were necessary to induce an axial moment Mx. General findings, for both non-aged and aged specimens, were reactive moments around the x‑axis during tensile testing, which were generated in all four groups 1–4 with the triple-stranded retainer wires.

**Fig. 4:**
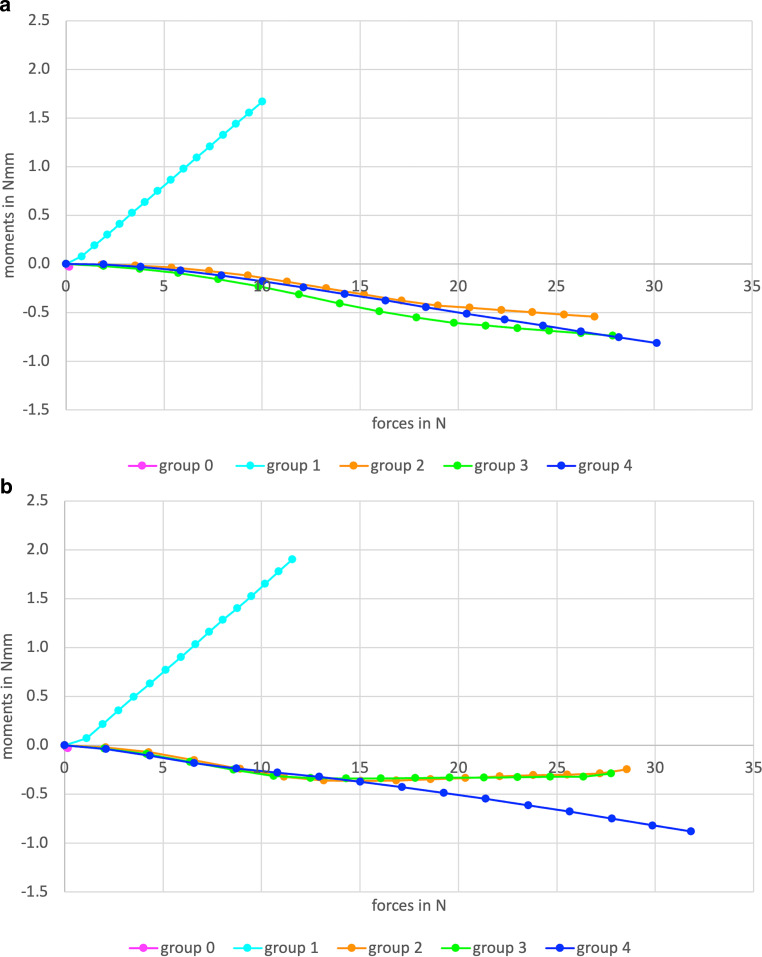
Force and moment values measured during tensile tests. Mean values of non-aged specimens (**a**) and mean values of artificially aged specimens (after storage in artificial saliva; 5000 cycles of thermocycling; **b**) Bei Zugversuchen ermittelte Kraft- und Drehmomentwerte. Mittelwerte nichtgealterter Proben (**a**) und Mittelwerte künstlich gealterter Proben (nach Lagerung im künstlichen Speichel; 5000 Zyklen Thermocycling; **b**)

**Fig. 5:**
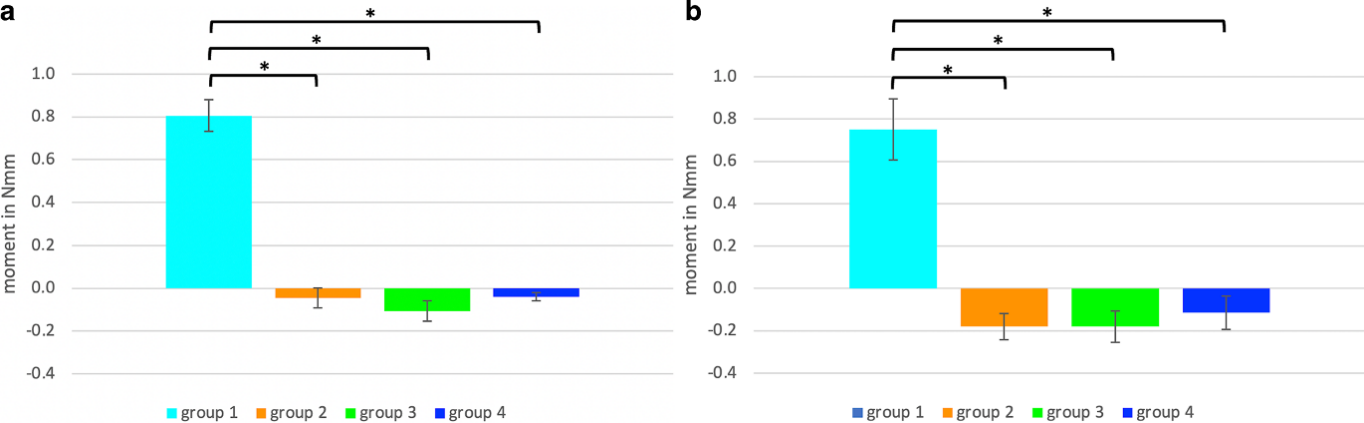
Column diagrams of mean moments occurring at tension force levels of 5.0 N of non-aged specimens (**a**) and aged specimens (**b**). The 95% intervals are indicated by error bars. * statistically significant differences (*p* < 0.05; *p*-values were Bonferroni-corrected) Säulendiagramme der resultierenden mittleren Drehmomente auf einem Kraftniveau von 5,0 N: nichtgealterte Proben (**a**) und gealterte Proben (**b**). Fehlerindikatoren zeigen das 95%-Konfidenzintervall. * statistisch signifikante Unterschiede (*p* < 0,05; *p*-Werte Bonferroni-korrigiert)

Fig. 4 provides a descriptive summary of all the force and moment components measured during the tensile tests for non-aged and aged specimens. In the control group (group 0) with the solid round stainless steel wire and an isolated composite–wire interface, mean forces and standard deviations of 0.1 ± 0.2 N and mean moments of 0.0 ± 0.0 Nmm were measured (pink dot in Fig. 4). Since no forces comparable to the other groups were generated here, this group cannot be used as a reference for comparing moments at specific force levels. Positive moments were measured in group 1 (triple-stranded wire, isolated composite–wire interface; turquoise lines in Fig. 4) with maximum moments of 1.7 Nmm (non-aged specimens) and 1.9 Nmm (aged specimens) for force levels of 10.0 N and 11.6 N, respectively. These Mx values pointed in opposite direction compared to those of groups 2–4: Negative moments were measured in groups 2–4 (triple-stranded wire, adhered composite–wire interface; orange, green and blue lines in Fig. 4). In group 1 the moment/force ratios were high, visualized by the steep slope of the turquoise curve reaching a moment of 0.5 Nmm already at a force of 3.4 N. The reactive moment reached a maximum value of 1.7 Nmm in the non-aged specimens (1.9 Nmm in aged specimens) at a force of 10.0 N/11.6 N (Fig. 5 turquoise lines). In contrast, the curves for wire groups 2–4 show clearly smaller slopes. Negative reactive moments of about −0.5 Nmm in these groups with adhered composite–wire interface were generated at a minimum of 18.0 N (groups 2–4, Fig. 4 orange, green, blue lines). Within the groups of adhered composite–wire interfaces, maximum Fx values for both non-aged and aged specimens indicated that the adhesive compound was strongest in group 4, followed by groups 3 and 2, leading to small differences in the moment/force ratio.

Fig. 5 summarizes results from the inferential statistical analyses at a force level of 5.0 N. Group 1 (triple-stranded wire, isolated composite–wire interface) showed the highest Mx values (0.7 Nmm ± 0.1 Nmm in the non-aged group and 0.8 Nmm ± 0.2 Nmm in the aged group). Positive moments of group 1 differed statistically significantly from zero, both for non-aged as well as for aged specimens (*p* < 0.001, Bonferroni-corrected). Moreover, they differed statistically significantly from the moment values of group 2 (both aged and non-aged *p* < 0.001; Bonferroni-corrected), group 3 (both aged and non-aged *p* < 0.001; Bonferroni-corrected), and group 4 (both aged and non-aged *p* < 0.001; Bonferroni-corrected). Moment values for the non-aged group 2 (Fig. 5a) showed no statistically significant difference from zero (*p* = 0.301, Bonferroni-corrected). However, negative moments for the other groups differed statistically significantly from zero (Fig. 5): for the aged group 2 (*p* < 0.001, Bonferroni-corrected), group 3 (aged *p* < 0.001, non-aged *p* = 0.001; Bonferroni-corrected), as well as group 4 (aged *p* = 0.049, non-aged *p* = 0.002; Bonferroni-corrected). No significant differences could be found between groups 2, 3, and 4 for both non-aged and aged specimens after Bonferroni-correction (*p* > 0.05).

**Fig. 6:**
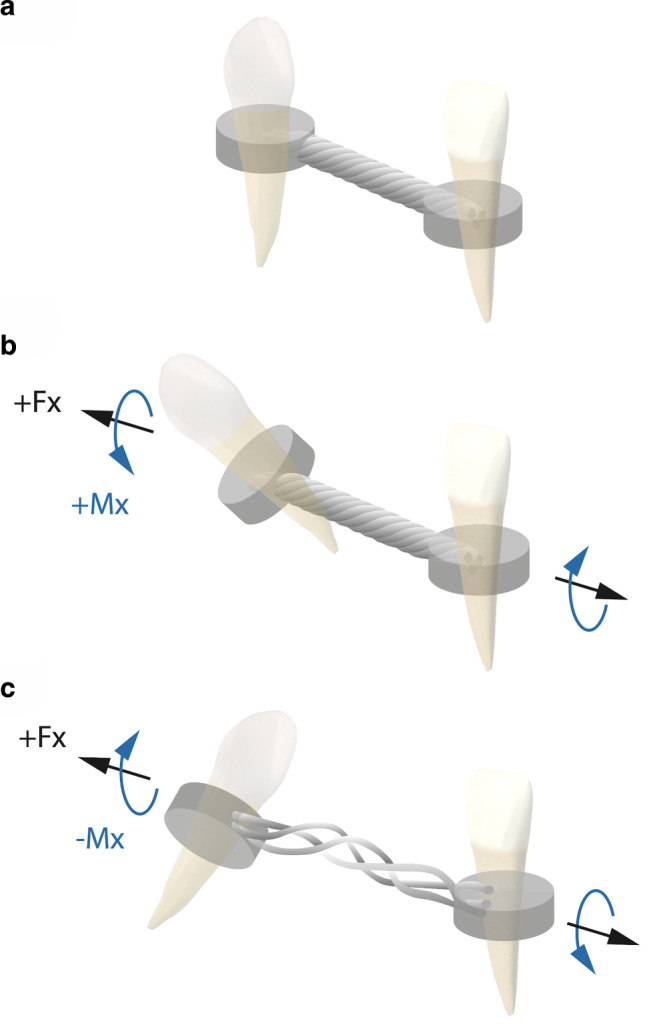
Generation of forces and moments at the two-tooth model depending on the different conditions at the composite–wire interface. The initial position of the two-tooth model with absence of tensile forces Fx shows consequently no rotational moments Mx (**a**). In presence of tensile forces, an isolated composite–wire interface (group 1) enables a relative movement between the wire and the composite. As a result, the application of tensile forces Fx (*black arrows*) may lead to a rotation of the left model tooth around the windings of the retainer resulting in a positive moment Mx (*blue arrow*) (**b**). In presence of tensile forces an adhered composite–wire interface (groups 2–4) does not enable any relative movement between the composite and the retainer wire. During application of tensile forces (*black arrows*), however, the triple-stranded retainer wire may unwind. In a left-handed retainer wire this may result in a clockwise rotation of the left model tooth and a negative moment Mx (*blue arrow*), respectively (**c**) Erzeugung von Kräften und Momenten im Zweizahnmodell in Abhängigkeit von den unterschiedlichen Bedingungen an der Komposit-Draht-Grenzfläche. In der Ausgangssituation des Zweizahnmodells ohne Applikation von Zugkräften Fx entstehen dementsprechend keine Drehmomente Mx (**a**). Bei Vorhandensein von Zugkräften ermöglicht eine isolierte Komposit-Draht-Grenzfläche (Gruppe 1) eine relative Bewegung zwischen Draht und Kunststoff. Bei der Applikation von Zugkräften (*schwarze Pfeile*) kann der linke Modellzahn um die Windungen des Retainerdrahtes rotieren, es entsteht ein positives Drehmoment (Mx, *blauer Pfeil,* **b**). Bei Applikation von Zugkräften ermöglicht eine feste, intakte Komposit-Draht-Grenzfläche (Gruppen 2–4) keine relativen Bewegungen zwischen Kunststoff und Retainerdraht. Bei Applikation von Zugkräften (*schwarze Pfeile*) kann sich der dreifach verseilte Retainerdraht entwinden. Bei einem linksgewundenen Retainerdraht kann dies zu einer Rotation des linken Modellzahns im Uhrzeigersinn und zu einem negativen Drehmoment Mx *(blauer Pfeil*) führen (**c**)

Based on these results, an explanatory model (Fig. 6) was developed to outline the generation of both positive (Fig. 6b) and negative moments (Fig. 6c) in presence of tensile forces.

## Discussion

The use of fixed retainers may cause unexpected changes and unwanted tooth movements such as the x‑ and twist effect. Although the x‑effect and twist effect have been described frequently in case reports [18, 23], their occurrence is generally considered to be rare. It has to be mentioned, however, that the number of bonded retainers and lifelong retention continues to increase [17] and the mentioned effects often emerge long term. As a long follow-up of patients is often difficult and produces costs, most patients terminate the control of retention appliances by orthodontists. Therefore, it is particularly important to better understand the underlying causes of inadvertent tooth movements.

This experimental in vitro study simulated the situation of two adjacent teeth under fixed retention exposed to external forces. Due to the geometrical conditions, such external forces may cause tensile forces between the two teeth as simulated in this experimental in vitro study. The results indicate that such tensile forces may actually cause rotational moments which may generate the inadvertent tooth movements observed in patients during retention. In the experimental setting, different parameters have been shown to determine the magnitudes and direction of the generated moments. More specifically, in the control group with isolated composite–wire interface and round solid retainer wire, very low tensile forces and rotational moments close to zero were measured during experimental increase of the intertooth distance by 0.15 mm. The low force magnitude may be explained by sliding of the round wire through the composite coating without resistance. The direction of the moment Mx in group 1 during tension was counterclockwise (defined as positive moment), due to the production of the investigated triple-stranded retainer wire with a left-handed winding direction. This effect may be compared with the corresponding movement of a screw-nut along the thread lead during application of tensile forces, as illustrated in Fig. 6. In contrast, a clockwise rotation, i.e. a negative moment Mx, may probably be caused by unwinding of the wire in groups 2–4. It seems that this effect occurs at higher force levels.

Based on these insights, however, it is still difficult to infer the direction of tooth rotation due to an x‑effect or twist effect. Retainer wires exist with both left-handed and right-handed windings, hence, producing differently directed moments. We suppose that the typically torqued position found in a terminal tooth of a retainer segment is more likely due to the effect described here for the isolated composite–wire interface (group 1, Fig. 6b) leading to rotation of the crown around the windings of the stranded retainer wire. We assume that in such x‑effects described in the literature, the composite–wire interface of the affected tooth was at least partially invisibly debonded or perhaps never fixed properly, equivalent to the isolation with petroleum jelly in group 1.

Some of the patients showing an x‑effect or twist effect show broken or at least loose adhesive compounds of the retainer wire, whereas in other cases the underlying reasons are unclear, as the retainer and its fixation to the teeth appears to be intact [18]. Our results suggest that a fixed retainer with intact, fixed adhesive compounds and a firm composite wire interface could only be based on an unwinding of the retainer wire itself (as illustrated in Fig. 6c, corresponding to groups 2–4). Although an unwinding of a multistranded wire has been discussed before [30], this mechanism can be assumed to be less frequent in the clinical setting because rather unrealistically high forces were required to unwind the intact wire in order to create such a moment.

In the clinic, oral dysfunctions and habits most likely generate the required tensile forces which are supposed to produce the x‑effect or twist effect. For instance, tongue dysfunction may lead to relatively high forces on the anterior teeth, as shown in untreated patients with protruded incisors [21]. Such linguobuccal forces may actually be transformed to tensile forces on the retainer–wire interfaces of connected adjacent teeth. In the context of the x‑effect or twist effect, this aspect has so far been largely neglected due to the short duration of tongue pressure [21, 23]. Some authors mention that the magnitude of resting tongue pressures may not be sufficient to cause bending of a fixed retainer wire [20, 23]. According to our findings, however, bending of the wire does not seem necessary to evoke moments and forces. Sifakakis et al. further confirmed that even small deflections of the retainer wire produce sufficiently high forces in the retainer segment [24]. The simulated oral forces were set at a force level of 5.0 N, corresponding to an oral loading of 500 g measured by Proffit et al. during swallowing [19]. The maximum bite force exerted on the incisors during biting can reach more than 100 N and might also cause unwanted direct mechanical deformities of the retainer wire [10, 11]. More likely, the stress of biting and chewing will additionally induce physiologic tooth movements that cause a horizontal displacement of the front teeth, turning out to be higher in the mandibular lateral incisors than in the canines [7, 24]. Notwithstanding the more general issue of external validity, our experimental setting was able to simulate physiological force levels sufficient to create positive and negative moments during the tensile tests.

Multistranded fixed lingual retainers are described as gold standard in orthodontic retention but favor the x‑effect [25, 27]. With regard to periodontal health, a highly flexible retainer is recommended as it allows more flexibility at the root–bone interface [22]. However, our results suggest that these two properties explain why inadvertent tooth movements are most often observed in patients with retainers made from multistranded wires [23]. Like on an inclined plane, tensile forces are converted into an axial moment via the screw windings of the multistranded wire. This effect is only possible, if the composite–wire interface is invisibly weakened within the composite, or was never properly fixed when inserted, allowing the tooth to rotate around the axis of the retainer wire (as illustrated in Fig. 6b, corresponding to group 1). Such bonding failure is almost impossible to be detected during routine control, since the outer composite–tooth interface seems intact.

Clinicians looking for options preventing the x‑effect or twist effect intuitively avoid round multistranded wires. For instance, Arnold et al. recommended the use of rectangular wires for minimizing the risk of inadvertent tooth movements. Actually, rectangular multistranded retainer wires allow more torque control which may prevent rotational moment around the wire windings though there is still a certain risk for wire unwinding [2]. Among the rectangular wires, a braided wire could be advantageous compared to the twisted wire [13]. Other authors prefer thicker 5‑stranded wires versus triple-stranded wires, but they do not succeed in completely avoiding inadvertent tooth movements [5, 14]. While no inadvertent tooth movements were found in patients wearing a retainer consisting of a solid round stainless steel wire [31], their use comes with material-specific disadvantages.

The biomechanical model (Fig. 6) explains not only the observed patterns of the x‑effect, but gives also an explanatory attempt for the twist effect. Depending on the winding path of the wire, we are able to predict the direction of generated moments. However, we cannot conclusively state whether the generated, opposing moments will cause an x‑effect or will affect the entire tooth block. The number of loosened compounds might be relevant for the difference between the development of the x‑effect or twist effect—affecting only one tooth or several teeth respectively. It seems likely that several compounds would have to be loose for an opposite inclination of contralateral canines [9], often found with the lower left canine inclined in the buccal direction and the right canine in the lingual direction [14]. An explanation of the twist effect should be further elaborated and tested. Respective experiments should be performed in a multiteeth model and could be verified in clinical observational studies. Finally, in addition to these outlined mechanical approaches, the multifactorial genesis of these phenomena, including anatomic conditions, should always be taken into account.

## Conclusion

This study shows that in fixed retainers, the conditions of the composite–wire interfaces may play a major role for generating inadvertent tooth movements, especially in combination with forces exerted by the tongue and lips. Rotational moments torqueing the (partially) debonded tooth around the retainer axis have to be distinguished from rotational moments in opposite direction due to unwinding of the wire. In this study, both mechanisms could be simulated in vitro, but the latter mechanism needed significantly higher force application and is therefore much less likely to occur in vivo. During routine examination of fixed retention, retainer wires that are apparently undamaged should be carefully checked and, if possible, the composite–wire interface tested to be intact. Caution should be given to tongue dysfunction that has not been eliminated during active treatment as this factor is supposed to increase the risk for inadvertent tooth movements. Based on our findings, round multistranded retainer wires without retention bendings at their ends are of higher risk in fixed retention.
